# Lentiviral Expression of Retinal Guanylate Cyclase-1 (RetGC1) Restores Vision in an Avian Model of Childhood Blindness

**DOI:** 10.1371/journal.pmed.0030201

**Published:** 2006-05-23

**Authors:** Melissa L Williams, Jason E Coleman, Shannon E Haire, Tomas S Aleman, Artur V Cideciyan, Izabel Sokal, Krzysztof Palczewski, Samuel G Jacobson, Susan L Semple-Rowland

**Affiliations:** 1Department of Neuroscience, University of Florida McKnight Brain Institute, Gainesville, Florida, United States of America; **2**Picower Institute for Learning and Memory, Massachusetts Institute of Technology, Cambridge, Massachusetts, United States of America; **3**Department of Ophthalmology, Scheie Eye Institute, University of Pennsylvania, Philadelphia, Pennsylvania, United States of America; **4**Department of Pathology, University of Washington, Seattle, Washington, United States of America; **5**Department of Pharmacology, School of Medicine, Case Western Reserve University, Cleveland, Ohio, United States of America; Moorfields Eye HospitalUnited Kingdom

## Abstract

**Background:**

Leber congenital amaurosis (LCA) is a genetically heterogeneous group of retinal diseases that cause congenital blindness in infants and children. Mutations in the *GUCY2D* gene that encodes retinal guanylate cyclase–1 (retGC1) were the first to be linked to this disease group (LCA type 1 [LCA1]) and account for 10%–20% of LCA cases. These mutations disrupt synthesis of cGMP in photoreceptor cells, a key second messenger required for function of these cells. The GUCY1*B chicken, which carries a null mutation in the retGC1 gene, is blind at hatching and serves as an animal model for the study of LCA1 pathology and potential treatments in humans.

**Methods and Findings:**

A lentivirus-based gene transfer vector carrying the *GUCY2D* gene was developed and injected into early-stage GUCY1*B embryos to determine if photoreceptor function and sight could be restored to these animals. Like human LCA1, the avian disease shows early-onset blindness, but there is a window of opportunity for intervention. In both diseases there is a period of photoreceptor cell dysfunction that precedes retinal degeneration. Of seven treated animals, six exhibited sight as evidenced by robust optokinetic and volitional visual behaviors. Electroretinographic responses, absent in untreated animals, were partially restored in treated animals. Morphological analyses indicated there was slowing of the retinal degeneration.

**Conclusions:**

Blindness associated with loss of function of retGC1 in the GUCY1*B avian model of LCA1 can be reversed using viral vector-mediated gene transfer. Furthermore, this reversal can be achieved by restoring function to a relatively low percentage of retinal photoreceptors. These results represent a first step toward development of gene therapies for one of the more common forms of childhood blindness.

## Abstract 2

Blindness associated with loss of function of retinal guanylate cyclase-1 in an avian model of Leber congenital amaurosis-1 can be reversed using viral vector-mediated gene transfer.

## Editors' Summary

**Background.:**

Leber congenital amaurosis (LCA) is the name of a group of hereditary diseases that cause blindness in infants and children. Changes in any one of a number of different genes can cause the blindness, which affects vision starting at birth or soon after. The condition was first described by a German doctor, Theodore Leber, in the 19th century, hence the first part of the name, and “amaurosis” is another word for blindness. About 20% of children with LCA have the most common type, called LCA1, which is caused by defects in a gene called *retinal guanylate cyclase (GUCY2D)* that is found on Chromosome 17. It is one of a group of genes that produce proteins that are important in determining how rods and cones—specialized light receptor cells at the back of the eye—respond to light, in particular, how they can return to the resting state after being stimulated by light. Defects in the gene leave the eye unable to respond to light, and so cause blindness. There is also damage to the receptor cells, which in turn causes a cellular breakdown of the retina—the light-sensitive tissue at the back of the eye. Gene therapy works by replacing a defective gene with a normal functional one, usually by packaging the normal gene into a harmless virus and injecting it into the affected tissue, in this case the eye.

**Why Was This Study Done?:**

Gene therapy is a promising therapy for diseases such as LCA, because the gene defect is known and the damaged cells are still alive but just not functioning properly. In addition, there is an animal model for LCA1, a strain of chickens with a mutation in the chicken *GUCY2D* gene: these chickens develop a disease very similar to that of humans and can be used to test treatments before they are tried out in humans.

**What Did the Researchers Do and Find?:**

They took a virus and put the normal *GUCY2D* gene into it and linked it to a reporter gene (that is a gene that can be used to check whether the first gene is present). They then injected this virus into eggs containing chick embryos that had the abnormal gene, and allowed the chicks to hatch normally. Of seven chicks injected, six exhibited sight, in contrast to untreated animals, which were blind. The improvement in the chicks' sight occurred despite the fact that only a relatively small amount of normal protein was made and that normal protein was present in only some of the retinal cells. The breakdown of the retinas in the treated chicks also appeared to be slower than in untreated chicks.

**What Do These Findings Mean?:**

This study shows that in theory it is possible to treat a form of childhood blindness caused by a photoreceptor gene defect by gene therapy. Because this study was done in chickens, many other steps need to be taken before it will be clear whether the treatment could work in humans. These steps include a demonstration that the virus is safe in humans, and experiments that determine what dose of virus would be needed and how long the effects of the treatment would last (the chicks were only studied for 6–7 weeks). Another question is whether it would be necessary (or even possible) to treat affected children before birth, or whether therapy in infants could restore their sight. In addition, the treatment would obviously only work for children who had this specific type of blindness, but many of the principles learned from studying LCA1 should be applicable to other types of LCA and possibly other types of inherited blindness.

**Additional Information.:**

Please access these Web sites via the online version of this summary at http://dx.doi.org/10.1371/journal.pmed.0030201.

• The Foundation for Retinal Research has detailed information on LCA

• The Foundation Fighting Blindness funds research into, and provides information on, many types of blindness, including LCA

• Contact a Family, a United Kingdom organization that aims to put families of children with illnesses in touch with each other, has a page of information on LCA

• RetNet—Retinal Information Network—is a comprehensive resource describing current knowledge of all inherited retinal diseases

• Fight for Sight funds student and post-doctoral fellowships as well as grant-in-aid to persons whose research focuses on curing and preventing blindness

• WebVision is a Web site that provides a comprehensive resource for understanding the organization and function of the retina and visual system

## Introduction

Vertebrate vision begins with absorption of light by visual pigments in retinal rod and cone photoreceptors. These activated pigments trigger a G-protein-coupled cascade that ultimately produces transient changes in the membrane potentials of the photoreceptor cells by modulating intracellular levels of cGMP, a key second messenger in this cascade. Rapid light-induced decreases in levels of cGMP lead to closure of cGMP-gated cation channels in the photoreceptor plasma membrane, hyperpolarization of the cell, and signaling to upstream neurons [1].

Precise regulation of cGMP synthesis and degradation, processes mediated by retinal guanylate cyclase–1 (retGC1) and cGMP phosphodiesterase (PDE), respectively, is critical to the function and health of photoreceptors (for review see [2]). Mutations in the *PDE6B* gene, which encodes the beta subunit of cGMP PDE, lead to abnormal increases in cGMP levels in photoreceptors, photoreceptor dysfunction, degeneration of these cells, and blindness in mouse [3,4], dog [5,6], and human [7]. Mutations in the *AIPL1* gene destabilize cGMP PDE and lead to photoreceptor dysfunction and a rapid retinal degeneration in mouse [8,9] and human [10]. Mutations in the gene encoding retGC1 that disrupt synthesis of cGMP lead to a loss of photoreceptor function that presents as blindness or severely compromised vision at birth followed by photoreceptor degeneration in chicken [11,12] and human [13–15].

The defective phototransduction second messenger system in homozygous PDE6-deficient mice was among the first targets for preclinical treatment using gene transfer [16–18]. The past decade has witnessed major progress in understanding disease processes in many animal models of human retinopathy, and this has escalated efforts to develop genetically based treatments (for review see [19]). To date, the most dramatic preclinical successes in the application of gene replacement strategies to treat inherited retinal disease have targeted defects in the retinal pigment epithelium [20–25] or in photoreceptor structure [26,27].

We have focused our efforts on developing a gene transfer approach to correct the defective phototransduction second messenger system of the GUCY1*B chicken. This animal carries a deletion rearrangement in the gene encoding retGC1 that produces a null allele [11]. The retinal disease exhibited by these animals models the human early-onset retinal degeneration known as Leber congenital amaurosis (LCA) type 1 (LCA1). The LCA1 gene, *GUCY2D* on human Chromosome 17p, was the first to be identified in this disease group and is estimated to account for 10%–20% of LCA patients [13–15]. In both the GUCY1*B chicken [12,28] and in patients with LCA1 [13], photoreceptor cell dysfunction precedes degeneration of these cells. To test our hypothesis that vector-mediated expression of retGC1 in the photoreceptors of GUCY1*B chickens can restore photoreceptor function and sight in these animals, we adopted an *in ovo* treatment strategy that allowed us to treat the retina prior to the appearance of pathology in these cells. In this report we describe a series of experiments that represent a first step toward our goal of developing treatments for a subset of LCA patients whose blindness is caused by *GUCY2D* mutations and for whom there is currently no cure [29].

## Methods

### Vector Construction and Lentiviral Vector Packaging

A lentiviral vector system was chosen for use in these experiments because the onset of expression of the transgene is rapid and stable throughout the course of retinal development [30,31], and these vectors are capable of carrying large gene constructs [32]. The construction of the pTYF-EF1α-PLAP vector has been described previously [33]. To construct the pTYF-EF1α-GC1-IRES-eGFP vector, the cDNA encoding bovine retGC1 was amplified in two parts from pSVL-ROS-GC1 [34] using the following core primers with ClaI linkers attached to the 5′ ends: (1) 5′-CCA TCG ATA GTT TAA ACG AGC CCC GGA CTT and 5′-GCC CAG CAC TGT TTC, and (2) 5′-GGC GAC GTC TTC AGT CT and 5′-CCA TCG ATG ACC CAG CCT CAC TTC C. The resulting fragments were subcloned into the ClaI site of a pBSII SK+ shuttle vector (Stratagene; http://www.stratagene.com) lacking the SacI site and were joined using the unique SacI site located within the amplified open reading frame to create pBS-bGC1. The complete guanylate cyclase–1 (GC1) cDNA was removed from pBS-bGC1 using ClaI and ligated into the ClaI site of the pTYF-EF1α-IRES-eGFP linker vector (http://www.mbi.ufl.edu/~rowland/vector.htm). The integrity of the GC1 coding region was confirmed by sequence analyses. Lentiviral vectors were packaged using previously described methods [33].

### In Vitro Analyses of Lentiviral Constructs and Virus

#### Transfection of DF1 cell cultures.

Before packaging the pTYF-EF1α-GC1-IRES-eGFP vector into lentivirus, we examined the function of the bicistronic transgene by monitoring expression of retGC1 and green fluorescent protein (GFP) in transiently transfected immortalized chicken fibroblasts (DF1 cells; ATCC; http://www.atcc.org). In vitro tests were performed using DF1 cells because they allowed us to test the functionality of the polio virus IRES sequence in the context of chicken cell translational machinery. Cells were seeded on tissue-culture-treated glass coverslips (Fisher Scientific; https://www1.fishersci.com) in 12-well plates and allowed to adhere overnight. When the cells reached ∼80% confluence, 0.5 ml of fresh medium was added to the cells, and 25 μl of the following transfection mixture was added to the cells: 3 μg of pTYF-EF1α-GC1-IRES-eGFP, 50 μl of plain Dulbecco's modified eagle medium, and 10 μl of Superfect reagent (Qiagen; http://www.qiagen.com). Following a 4-h incubation period, fresh medium was added to each well, and the cells were maintained for 48 h before processing for immunocytochemistry.

#### Immunocytochemistry.

Forty-eight hours after transfection, cells were fixed using 4% paraformaldehyde for 5 min. Cells were then incubated in blocking buffer (10% goat serum in phosphate-buffered saline [PBS]) for 30 min. A rabbit polyclonal antibody to GC1 (GC2; gift from A. Yamazaki) was diluted 1/333 in PBS containing 1.0% bovine serum albumin and 0.3% Triton X-100, and added to the cells, which were then incubated overnight at 4 °C [35]. Primary antibody detection was performed using goat anti-rabbit secondary IgG tagged with the Alexa-594 fluorophore (1/500 in PBS) (Molecular Probes, Invitrogen; http://probes.invitrogen.com). Immunostained cells were counterstained with 4′,6′-diamino-2-phenylindole.

#### Transduction of TE671 cell cultures.

After packaging the pTYF-EF1α-GC1-IRES-eGFP vector into virus, the function of the encoded GC1 enzyme was examined by transducing human-derived TE671 cells (ECACC; http://www.ecacc.org.uk) with the virus and then assaying these cells for GC1 activity. TE671 cells were seeded into the wells of a 24-well plate and grown overnight to ∼90% confluence. On the following day, the cells in each well were infected with 0.3 ml of fresh medium containing approximately 10^6^ transducing units (TUs) of either EF1α-GC1-IRES-eGFP or EF1α-eGFP virus. After 24 h, the cells were trypsinized, seeded into T-25 flasks, and maintained by passaging two times a week. The cells were harvested for use in GC1 activity assays after ∼20 passages. Cell pellets were snap-frozen in liquid nitrogen and stored at −80 °C until use.

#### GC1 activity assays.

Washed rod outer segment membranes prepared from fresh bovine retinas [36] and cell membranes of TE671 cells infected with either EF1α-GC1-IRES-eGFP or EF1α-eGFP virus were reconstituted with recombinant guanylate cyclase activating protein–1 (GCAP1) and assayed for GC1 activity as previously described [37]. Calcium concentration was calculated using the computer program Chelator 1.00 [38] and adjusted to higher concentrations by increasing the amount of CaCl_2_. All assays were repeated at least twice.

### Experimental Animals

A breeding colony of GUCY1*B Rhode Island Red (RIR) chickens [39] is maintained at the University of Florida and is cared for in accordance with National Institutes of Health guidelines. Chickens were raised from hatching under 12-h-on/12-h-off cyclic lighting. Three experimental groups were included in this study: treated GUCY1*B chickens (*n* = 7), untreated GUCY1*B chickens (*n* = 3), and wild-type, untreated RIR chickens (*n* = 5).

### Vector Injection and Hatching Procedures

Prior to injection, on day 0, eggs were washed with a blend of quaternary detergents (Biosentry RCL; http://www.antecint.co.uk/main/biosentry-products27.htm) in a water bath preheated to 43 °C. Dried eggs were then incubated without rotation on their sides at 37.5 °C and 60% humidity. On embryonic day 2 (E2) (stage 10–12) [40], the position of the embryo was determined using an egg-candling light and a 5- to 7-mm opening was made in the eggshell overlying the embryo without disturbing the membrane adjacent to the shell. Sterile PBS (50–100 μl) was applied to the exposed membrane, which was then removed. Viral vector was delivered to the neural tube using a pulled glass capillary needle (tip diameter ∼40 μm) held by a micromanipulator and connected to a manual hydraulic microinjector (Sutter Instrument; http://www.sutter.com). Approximately 0.5 μl of vector containing 0.03% fast green dye was slowly injected into the ventricular space of the developing neural tube with the aid of a dissecting microscope (2.5×). The opening in the eggshell was then sealed with Parafilm M (Fisher Scientific) using a warmed spatula to adhere the film to the shell. Post-injection, eggs were incubated upright, large end up, at 37.5 °C and 60% humidity and were slowly rocked three times per day (Sportsman Incubator; GQF Manufacturing; http://www.gqfmfg.com). At E18, eggs containing surviving embryos were moved to a second incubator for hatching. From E18 through hatch, eggs were incubated on their sides, were stationary, and were maintained at 37.5 °C and 68%–70% humidity (AV-2 Precision Parrot Incubator, Avey Incubator; http://www.aveyinc.com). Post-hatch chicks were reared for 3 to 5 d in an Octagon TLC-4 brooder (Brinsea; http://www.brinsea.com) and then transferred to the University of Florida animal housing unit.

### Behavioral Analyses

Animals were tested for the presence of both reflexive and volitional visual behaviors every 3 to 10 d for 6 wk. Reflexive visual responses were assessed using an optokinetic nystagmus (OKN) paradigm. The OKN reflex is driven primarily by visual stimuli processed by the peripheral regions of the retina. The reflex is manifest as a compensatory head movement of the animal in an attempt to fixate a moving stimulus. The stimuli were two high-contrast vertical square wave gratings with spatial frequencies of 0.065 or 0.26 cycles·degree^−1^ (bar widths of either 5 cm or 1.25 cm, respectively). Stimuli were presented in the form of a rotating drum (average speed 14.6 rpm) and were evenly illuminated from above (60-W incandescent bulb). The animals were held stationary in the center of the drum while it was rotated in both clockwise and counterclockwise directions. The behavior of the animals was also examined when the stimulus was not moving, serving as a reference point for evaluation of the behaviors elicited by the moving stimuli. A positive OKN response was characterized by a smooth head movement in the direction and at the speed of stimulus rotation followed by a rapid head movement in the opposite direction. Behavior was recorded (Nikon Coolpix Digital Camera and Video Recorder; http://www.nikon.com) and analyzed for the presence of OKN responses using a zero to three scoring system: zero, no OKN response; one, inconsistent or unidirectional responses to the lower spatial frequency grating; two, consistent bidirectional responses to the lower spatial frequency grating; three, consistent bidirectional responses to the higher spatial frequency grating.

Volitional visual responses were assessed by placing animals in a testing environment that contained novel visual stimuli including colored candies, aluminum foil, and metal objects. Volitional visual behavior is driven primarily by stimuli processed by the central/foveal regions of the retina. The animal's interest in its visual environment drives this behavior. Animals were exposed to the testing environment for periods of approximately 3 to 5 min, and behavior was video-recorded and analyzed using a zero to three scoring system: zero, no visual behavior, with random head drift; one, evidence of orientation to surroundings, with no random head drift detected; two, head movements coordinated with the presence and/or movement of visual stimuli; three, pecking of visual targets and stimuli.

### Electroretinograms

The pupils of dark-adapted (>12 h) animals were dilated (repeated topical administration of vecuronium bromide, proparacaine HCl, benzalkonium chloride, and tropicamide) over a 30-min period prior to the recordings. The animals were then anesthetized using a mixture of ketamine HCl (10 mg/kg) and xylazine (2.5 mg/kg) delivered intramuscularly. A quarter of the initial dose was given as needed during the recording session to maintain anesthesia. Anesthetized animals were placed in a supine position with their heads resting on a head holder. Full-field electroretinograms (ERGs) were recorded from the right eye of each animal using custom-made contact lens electrodes (Hansen Ophthalmics, Iowa City, Iowa, United States). An eyelid speculum was used, and the electrode was held in place by a stereotaxic apparatus. A platinum needle placed in the skin above the eye served as reference. ERGs were recorded using a commercially available ganzfeld- and computer-based system (ColorDome and Espion Console, Diagnosys; http://www.diagnosysllc.com). Recordings began with dark-adapted ERG luminance-response functions elicited with increasing intensities of white flashes (−3.2 to +0.8 log cd·s·m^−2^; 0.5 log unit steps; 2-s interstimulus interval; digital filter disabled). For low-intensity stimuli, 4–10 responses were recorded and averaged. For the highest-intensity stimuli, 2–6 responses (>15-s interstimulus interval) were recorded. Upon completion of the dark-adapted stimulus series, the animals were light-adapted to a 30-cd·m^−2^ white background, and the ERGs elicited by 29-Hz flicker stimulation (white; +0.8 log cd.s·m^−2^) were recorded and averaged (20 responses). In some cases, 250–750 responses were recorded to detect submicrovolt-amplitude flicker ERGs. The amplitudes of the ERG waveforms were measured conventionally: a-waves were measured from baseline to the trough, b-waves were measured from baseline or from the a-wave trough to the positive peak, and 29-Hz flicker amplitudes were measured from trough to peak.

### Tissue Collection for Histological and Molecular Analyses

The animals were sacrificed within 1 wk of the last behavioral testing period. Animals were anesthetized with an intramuscular injection of ketamine (16 mg/kg) and euthanized using a protocol approved by the University of Florida Institutional Animal Care and Use Committee. The eyes were then rapidly enucleated and the anterior segment and vitreous body of each eye were dissected away. The posterior eye cup of the right eye was placed in 4% paraformaldehyde and fixed overnight at 4 °C. Following fixation, one half of the bisected right eye cup was processed for semi-thin plastic histology, and the other half was processed for frozen sectioning and extraction of genomic DNA. The left eye cup was bisected along the superior/inferior midline axis, and equal portions of the retina/retinal pigment epithelium/choroids were removed, placed in sealed tubes, rapidly frozen in liquid nitrogen, and stored at −70 °C until use for RNA analyses.

To permit use of the right and left retinas of treated animals in our analyses, we determined if delivery of the virus via the neural tube produced similar transduction percentages in both eyes. Three embryos were injected with 0.5 μl of pTYF-EF1α-PLAP lentivirus (5 × 10^9^ TUs/ml) to compare the pattern and percent transduction of left and right retinas. On E10, the embryos were sacrificed and the retinas were processed for PLAP staining and analyzed as described previously [30,33]. The patterns and percent transduction values for the left and right retinas of individual animals were similar (41%, 39%; 18%, 13%; 9%, 15%). The variability observed in the percent transduction between animals was dependent on the quality of the embryonic injection and decreased as percent transduction increased. No striking interocular asymmetry was observed in the percent viral transduction within individual animals.

### Retinal Immunohistochemistry and Light Microscopy

Following fixation in 4% paraformaldehyde, the right eye of each animal was bisected along the superior/inferior midline axis: the temporal portion of each eye cup was processed for immunohistochemical analyses while the nasal portion was processed for semi-thin plastic analyses. Tissues designated for immunohistochemical analyses were cryoprotected by soaking overnight in a 30% sucrose (wt/vol)–PBS solution, sectioned (14 μm), and stored at −30 °C until stained. Prior to immunostaining, the tissues were dried overnight, rinsed in PBS, and permeabilized and blocked for 1 h in PBS containing 0.3% Triton X-100, 1% bovine serum albumin, and 10% goat serum. GFP was detected using a polyclonal antibody (generously provided by W. Clay Smith, University of Florida, Gainesville, Florida, United States) diluted 1:500 in primary dilution buffer (PBS containing 1% bovine serum albumin and 10% goat serum). Sections were incubated with the primary antibody overnight at 4 °C. The primary antibody was visualized by labeling with a goat anti-rabbit IgG secondary antibody tagged with the Alexa-488 fluorophore (Molecular Probes, Invitrogen) diluted 1:500 in primary dilution buffer. Sections were counterstained with 4′,6′-diamino-2-phenylindole, mounted in Gel/Mount aqueous media (Biomeda;http://biomeda.com), coverslipped, and sealed with Permount resin. Tissues designated for semi-thin plastic analyses were dehydrated through a graded series of ethanol solutions (50%, 70%, and 80%) and embedded in JB-4 Plus (Electron Microscopy Sciences; http://www.emsdiasum.com/microscopy) using the manufacturer's protocol. Plastic embedded tissues were sectioned (1.5 μm), stained with 1% toluidine blue in 1% (wt/vol) sodium borate, and coverslipped using Permount resin. Immunohistochemical and plastic sections were examined and photographed using a Zeiss Axioskop 2 plus (http://www.zeiss.com) fitted with a Spot image acquisition system (Diagnostic Instruments; http://www.diaginc.com).

The immunostained and semi-thin plastic sections were analyzed to obtain information regarding the extent of viral transduction and the effects of the treatment on retinal morphology, respectively. We were unable to estimate the percent viral transduction of the retina by examining flat-mounted retinas because GFP expression was too low to allow direct visualization of the transduced cells. Low protein expression is frequently observed from the second cistron of IRES-based bicistronic expression cassettes in vivo [41]. Information about the distribution of transduced cells was obtained by analyses of GFP immunofluorescence of serial sections cut along the superior/inferior axis of the right eyes of the treated animals. Immunostained serial retinal sections were each divided into four regions relative to the optic nerve and were designated far superior (FAR SUP), superior (SUP), superior optic nerve (SUP ON), and inferior optic nerve (INF ON). Each region was assigned a percent transduction score by an independent observer that was based on the number of GFP-positive cells within the region. These scores were plotted to yield 3-D graphic representations of transduction across the 500-μm retinal expanse using SigmaPlot v8.0 (Systat; http://www.systat.com). To assess the effects of treatment on retinal morphology, the total width of the retina extending from the outer limiting membrane to the ganglion cell layer was measured at 100-μm intervals within each of the four retinal regions. Measurements were made from digital images of representative semi-thin retinal sections using Adobe Photoshop v7.0 (Adobe; http://www.adobe.com). For each region, the retinal widths of the treated retinas were expressed as percent change relative to the average width of the corresponding regions in age-matched GUCY1*B untreated retinas.

### Genomic and Reverse Transcription PCR

Genomic DNA was extracted from 25 mg of retina/pigment epithelial tissue taken from the right eye that had been fixed and cryoprotected but not sectioned. The tissues were soaked in PBS to remove the sucrose, and DNA was extracted from the tissue using a DNAeasy kit (Qiagen). Total RNA was extracted from retina/pigment epithelium tissue that had been removed from the left eye and stored at −70 °C. The frozen tissue was pulverized under liquid nitrogen, and RNA was extracted using an RNeasy kit (Qiagen) according to the manufacturer's recommended protocol that included treatment of the RNA samples with RNA-free DNAse to remove trace quantities of genomic DNA. Known copy numbers of pTYF-EF1α-GC1-IRES-eGFP plasmid DNA ranging from 3,000 to 300,000 copies were prepared and amplified in parallel with the genomic DNA samples.

The plasmid DNA standards, genomic DNA, and total RNA were amplified using primers that spanned the IRES-eGFP elements present in the lentiviral transgene (sense: 5′-TTT CCC CGG TGA TGT CGT; antisense: 5′-GCC GGT GGT GCA GAT GAA). The reverse transcription (RT)–PCR analyses included amplification of chicken β-actin mRNA (sense: 5′-TGC TGC GCT CGT TGT TG; antisense: 5′-GTC ACG GCC AGC CAG AT) to control for the quality and quantity of RNA in each sample and the efficiency of the RT reaction. PCR amplification of standard and genomic (0.5 μg of DNA template) DNA was carried out in 50-μl reactions. RT-PCR reactions were carried out in two steps. Total RNA (1 μg) was reverse transcribed in a 50-μl reaction volume. Aliquots of the RT reaction were then amplified for GC1 (15 μl) and β-actin (3 μl) transcripts in separate reactions (50-μl final volume). Components for the genomic and RT-PCR reactions were obtained from an Ampli*Taq* Gold RT-PCR kit (Applied Biosystems, http://www.appliedbiosystems.com). The reaction parameters used to amplify the GC1 transgene and its transcript were 95 °C for 2 min; 35 cycles of 95 °C for 1 min, 61 °C for 1 min, and 72 °C for 1 min; 72 °C for 10 min; and 4 °C soak. The reaction parameters used to amplify the β-actin transcript were the same as above except that amplification was carried out for 30 cycles. Aliquots (20 μl) of the PCR reactions were separated on a 1% agarose gel containing 32 nM ethidium bromide, and photographed and analyzed using a Gel Doc 1000 system and Quantity One software (Bio-Rad; http://www.bio-rad.com). The RT-PCR analyses were repeated three times. For each RT-PCR trial, the quantity of transgene mRNA in each sample was normalized to the average amount of β-actin mRNA present in the samples.

The number of integrated vector transgenes in the genomic DNA extracted from the treated tissues was estimated by comparing the amount of product obtained in these reactions to that obtained from PCR amplification of known copy numbers of the respective plasmid DNA. The genomic DNA and standard reactions were amplified and analyzed under identical conditions and were repeated three times. When imaging the PCR gels, care was taken to ensure that the signals were below saturation. The intensity values for the standards obtained from three independent trials were plotted and analyzed using SigmaPlot 8.0 (Systat). The number of vector transgene copies present in the genomic DNA samples was calculated using the equation for the best-fit sigmoid curve. These values were converted to vector transgene copies per genome copy (transgenes/genome) using the value of 380,000 genome copies/0.5 μg of chicken DNA [42].

### Statistical Analyses

The morphological data obtained from the semi-thin plastic sections were analyzed using a one-sample *t*-test to determine if the mean percent change in retinal thickness of treated animals relative to untreated GUCY1*B age-matched controls was significant in the four retinal regions examined.

## Results

### In Vitro Characterization of the pTYF-EF1α-GC1-IRES-eGFP Vector

**Figure 1:**
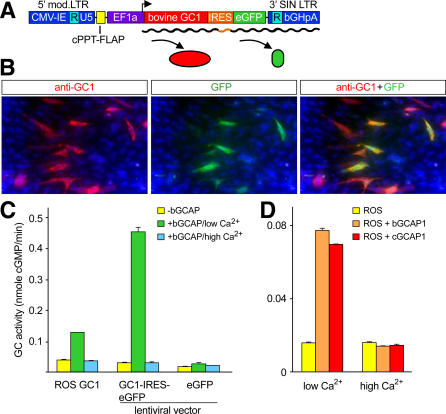
In Vitro Analyses of the Function of the pTYF-EF1α-GC1-IRES-eGFP Vector and Virus (A) Diagram of the bicistronic vector indicating production of bovine GC1 and GFP proteins from a single transcript. (B) DF1 cells transiently transfected with the vector and subsequently analyzed for expression of bovine GC1 and GFP. Cells immunostained with antibody recognizing bovine GC1 also expressed GFP. (C) Comparison of GC1 activity measured in bovine rod outer segments (ROS), TE671 cells transduced with EF1α-GC1-IRES-eGFP virus, and TE671 cells transduced with the control virus, EF1α-eGFP. Activity was assayed in the presence or absence of bovine GCAP1 under both high- and low-calcium conditions and is expressed as nanomoles of cGMP produced per minute. (D) Examination of ability of chicken GCAP1 to activate bovine GC1 under physiological conditions. GC1 activity was measured in preparations of bovine rod outer segments in the presence and absence of bovine GCAP1 and chicken GCAP1 under high- and low-calcium conditions. All assays were conducted in triplicate. Bars represent mean ± SEM.

Prior to packaging the pTYF-EF1α-GC1-IRES-eGFP vector, the function of the transgene was examined by transfecting DF1 cells with the plasmid and immunostaining the cells for GC1. DF1 cells transfected with pTYF-EF1α-GC1-IRES-eGFP (Figure 1A) stained positively for bovine GC1 and in many cases exhibited GFP fluorescence indicating that both cistrons of the bicistronic transgene were being expressed (Figure 1B). Not all GC1-positive cells were GFP-positive. The low levels of GFP protein in these cells is consistent with several reports of reduced protein expression from cistrons located downstream of IRES elements [41]. The pTYF-EF1α-GC1-IRES-eGFP vector was packaged into virus, and the function of the transgene following integration was examined using TE671 cells. TE671 cells infected with EF1α-GC1-IRES-eGFP lentivirus expressed GFP (data not shown). Biochemical analyses of membrane fractions prepared from these cells revealed that the GC1 protein encoded by the transgene exhibited robust GCAP1-dependent activity that was modulated by calcium in a physiological manner (Figure 1C) [43]. Since we used cDNA encoding bovine GC1 in our vector constructs, we also conducted experiments to examine the ability of chicken GCAP1 to activate bovine GC1 under physiological conditions. The results of these analyses showed that activation of bovine GC1 by chicken GCAP1 is calcium-dependent and that the level of activation is comparable to that induced by bovine GCAP1 (Figure 1D).

### In Vivo Lentiviral Treatment Restores Optokinetic and Volitional Visual Behaviors

**Figure 2:**
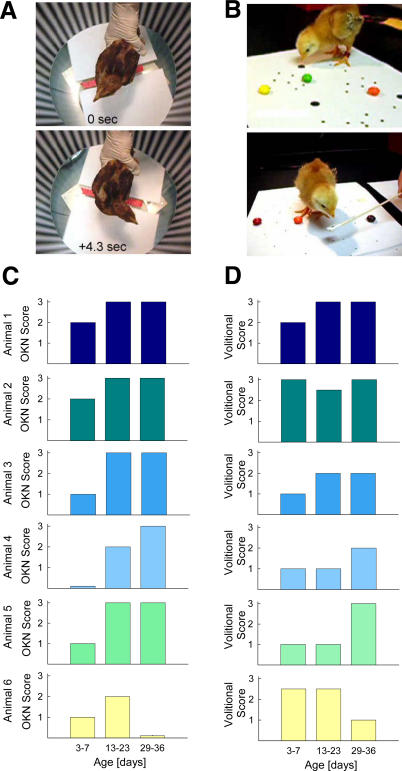
Optokinetic and Volitional Behavior Tests Indicate Treatment Efficacy (A) Optokinetic reflex (OKN) exhibited by 21-d-old, wild-type RIR chicken. Two frames of Video S1 (4.3 s between frames) are shown illustrating the head movement observed in birds in response to counterclockwise rotation of the 0.26-cycles·degree^−1^ (bar width = 1.25 cm) stimulus. (B) Volitional visual behavior exhibited by treated GUCY1*B animal 2 on day 7. Two frames of Video S4 are shown illustrating the animal's abilities to perceive and peck at objects within its visual field. (C and D) Graphic summaries of the behavioral test results for the six treated animals that exhibited sighted behavior. (C) Graphs showing the optokinetic scores (0–3) for the six treated animals as a function of age. (D) Graphs showing the volitional behavior scores (0–3) for the six treated animals as a function of age. The graphs are color coded by animal.

Seven GUCY1*B embryos were treated with EF1α-bGC1-IRES-eGFP lentivirus. Of the seven treated animals, six exhibited varying degrees of sighted behavior over the course of the 4- to 5-wk testing period. One treated animal failed to exhibit sight as measured by behavioral testing. We conducted electrophysiological tests on this animal, but did not process its retinas for molecular or morphological analyses. The visual capabilities of the six treated animals exhibiting sight were assessed by examining their optokinetic reflexes (Figure 2A; Video S1) and volitional visual behaviors (Figure 2B; Video S2). These tests allow assessment of visual function subserved by different regions of the retina and central nervous system. The OKN test is dominated by the function of the peripheral retina while the volitional test is dominated by the function of the central/foveal retina [44–46]. All treated, sighted animals exhibited robust OKN responses to the two different spatial frequencies tested (Video S3), with an overall group mean score of 2.23. Volitional, sight-directed pecking behavior was observed in treated animals as early as 3 d post-hatching. They also exhibited high levels of exploratory behavior and were able to peck at a variety of objects within their visual fields (Video S4). The mean volitional behavior score for the six treated animals over the entire study period was 2.05. On the final day of testing, five of the six treated animals received OKN scores of three and volitional behavior scores of either two or three. The visual behavior scores of the remaining animal dropped during the last week of the study. At the time of sacrifice, this animal did not show any evidence of volitional sight. Wild-type and untreated GUCY1*B animals received mean scores of 3.0 and zero, respectively, on both the OKN and volitional behavior tests. A summary of the vision test results for the six treated animals exhibiting sight is shown in Figure 2C and 2D.

### Retinal Electrophysiology Also Indicates Treatment Restores Function

**Figure 3:**
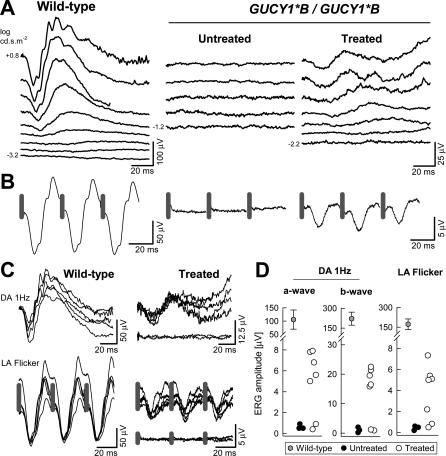
Retinal Electrophysiology Rescued in Treated Eyes (A) Comparison of dark-adapted ERGs in response to increasing intensities of light in a GUCY1*B/GUCY1*B animal injected with EF1α-bGC1-IRES-eGFP (“treated”) compared to a control animal (“untreated”). ERGs in the untreated animal are nondetectable, in contrast to the sizeable ERGs evoked in the treated animal. Results from a wild-type animal are shown in the left column for comparison. (B) Light-adapted 29-Hz flicker ERGs in the same animals as shown in (A) demonstrate restoration of responses after treatment. (C) Overlapping waveforms are ERGs elicited by 0.8 log cd·s·m^−2^ white flashes presented in dark-adapted (“DA 1 Hz”) and light-adapted (“LA Flicker”) states in all treated animals compared to wild-type controls. Functional rescue was observed in five (top waveforms) of the seven treated animals, whereas two (bottom waveforms) showed responses indistinguishable from noise. (D) Summary parameters of dark-adapted photoreceptor (a-wave) and post-photoreceptor (b-wave) function, as well as light-adapted flicker amplitude in treated animals as compared to untreated and wild-type animals. Five of seven treated animals showed amplitudes substantially larger than untreated animals but smaller than wild-type controls (gray symbols; mean ± standard deviation).

Electroretinography conducted under both dark- and light-adapted conditions was used to assess photoreceptor-mediated retinal function in wild-type, untreated GUCY1*B, and treated GUCY1*B chickens 3–4 d prior to sacrifice. All animals were 31–37 d of age at the time of testing. ERGs in wild-type and untreated GUCY1*B chickens differed dramatically (Figure 3A). Dark-adapted wild-type animals had ERG responses with a-wave (photoreceptor origin) and b-wave (bipolar cell origin) components that increased in amplitude with increasing stimulus intensity (Figure 3A, left). Dark-adapted, untreated GUCY1*B animals failed to produce measurable ERG responses to stimuli of even the highest intensities (Figure 3A, middle). Light-adapted wild-type chickens had large-amplitude cone-mediated ERG responses when presented with a 29-Hz flicker stimulus in the presence of rod-desensitizing background illumination (Figure 3B, left). Light-adapted, untreated GUCY1*B animals had no detectable flicker ERG responses (Figure 3B, middle). Treatment of GUCY1*B animals with EF1-bGC1-IRES-eGFP lentivirus restored retinal function (Figure 3A and 3B, right). Five of seven (71%) treated animals had ERG responses to single flashes under dark-adapted conditions and to flickering stimuli under light-adapted conditions. The shapes of these responses were similar to those generated by wild-type animals but with lower amplitudes. Two of the treated animals had no detectable responses (Figure 3C). The amplitudes of the ERG a-waves in the five responding animals (6.6 ± 1.3 μV [mean ± standard deviation]) were 6% of the wild-type response (105.8 ± 36.0 μV), suggesting that phototransduction had been restored in a subset of photoreceptors. The two treated animals that did not exhibit visual behavior failed to produce ERGs that were distinguishable from noise (Figure 3D).

### The Number of Integrated Transgenes per Genome Is Related to Treatment Efficacy

**Figure 4:**
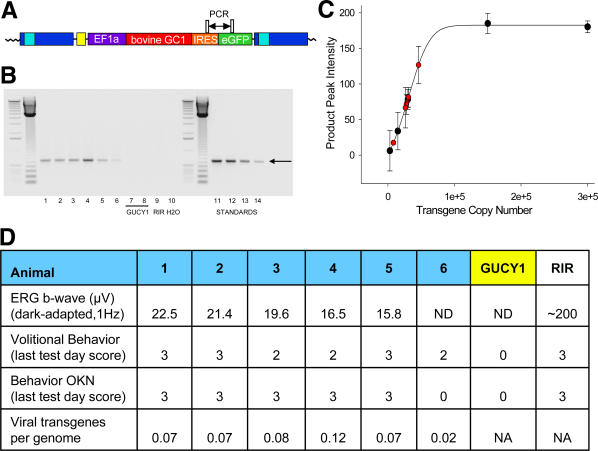
Quantitative Genomic PCR Estimate of Integrated Viral Trangenes per Genome (A) Diagram of integrated viral transgene and location of PCR primers spanning the IRES-eGFP junction that were used to amplify the transgene. (B) Ethidium-bromide-stained gel showing 638-bp product (indicated by arrow) amplified from the experimental and standard reactions. Lanes 1–6 contain samples amplified from the six treated animals exhibiting restored sight. No product was observed in untreated GUCY1*B (lanes 7 and 8) or wild-type (lane 9) chickens. A blank water control is shown in lane 10. Lanes 11–14 contain product amplified from 300,000, 150,000, 30,000, and 15,000 copies of the transducing vector. DNA size ladders, 1 kb and 123 bp, were run to the left of the samples and standards. (C) Plot of amount of product obtained from the standard PCR reactions (black) best fit with a sigmoid curve (*f* = *y*_0_ + *a*/(1 + exp(−(*x −x*_0_)/*b*))*^c^* with *r* ^2^ = 0.999 . The products obtained from each experimental sample (red) fell within the linear portion of the amplification curve, and the number of transgenes present in each sample was calculated using the equation for the best-fit curve. Individual values plotted are mean ± SEM (*n* = 3). (D) Comparisons of integrated viral transgenes per genome, dark-adapted ERG b-wave amplitudes (1 Hz), volitional behavior scores, and optokinetic reflex scores. The data for the ERG and behavioral tests were collected at the end of the 6-wk study period. The results for the six treated animals exhibiting sight and for age-matched untreated GUCY1*B and wild-type RIR animals are shown. NA, not assayed; ND, not detected.

Quantitative genomic PCR was carried out on DNA extracted from the right eyes of the six treated animals that exhibited visual behavior to obtain a measure of the efficiency of the viral treatment. The retinas of the seventh animal, which did not exhibit evidence of sighted behavior following treatment, were not processed for molecular or morphological analyses. The DNA samples were analyzed on three different days, and each reaction set included amplification of DNA standards containing known copy numbers of the transducing vector. The primers were designed to amplify a 638-bp product that spanned the IRES-eGFP elements within the transgene (Figure 4A). The lentiviral transgene was detected in all six samples obtained from the treated animals; no product was amplified from untreated GUCY1*B, wild-type RIR, or water control reactions (Figure 4B). The amount of PCR product obtained in all experimental samples fell within the linear portion of the standard amplification curve (Figure 4C) used to calculate the number of copies of the viral transgene within each sample. The estimated number of integrated viral transgenes per genome in the retinas of the six treated animals ranged from 0.12 to 0.02 with a mean value of 0.07 ± 0.01(mean ± standard error of the mean [SEM]). A comparison of the integrated viral-transgene-per-genome values and the ERG and behavioral data obtained from the experimental and control animals at the end of the 6-wk study is shown in Figure 4D. A noteworthy finding is that the animal with the lowest transgene copy number was the animal that toward the end of the study scored poorly on the visual behavior tests and had no measurable ERG responses.

### RT-PCR Analyses and GFP Immunostaining Confirm Transgene Expression

RT-PCR analyses were carried out to examine transgene expression in the retinas of treated animals. Amplification of aliquots of reverse-transcribed RNA revealed that the transcript derived from the viral transgene was present in all six of the treated retinal samples. The 638-bp PCR product for the transgene was not detected when the RT step was omitted from the procedure, nor was it detected in GUCY1*B untreated or wild-type retina samples. All samples contained approximately equal amounts of β-actin mRNA as determined by the staining intensity of the 538-bp β-actin product. The amount of transgene and β-actin transcript in each sample was quantified, and the amount of transgene mRNA was normalized to the average amount of β-actin mRNA detected across all samples run in that particular assay. The relative amount of transgene mRNA present in each of the experimental samples, determined from three independent assays, ranged from a high of 144 to a low of 19, with a mean value of 94 ± 12 (mean ± SEM). In general, transgene mRNA levels correlated with estimates of the number of integrated transgenes per genome within animals. Variations in these measures were likely due to sampling variability arising from the random distribution of transduced cells and use of different tissue samples for these analyses.

**Figure 5:**
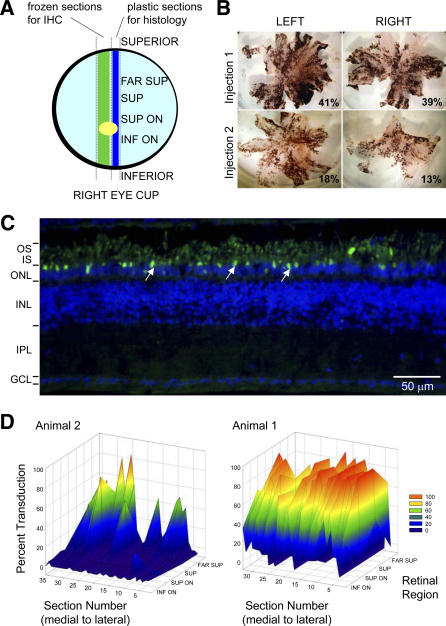
Immunohistochemical Analyses of GFP Expression in Treated Retinas (A) Schematic of the right eye cup that shows how the retina was apportioned for analyses. The right eye of each animal was bisected along the vertical meridian. One half was processed for immunohistochemical (IHC) analyses and the other for detailed histological analyses. The retinal sections were divided into four regions (FAR SUP, SUP, SUP ON, and INF ON) to simplify analyses. (B) Flat-mount retinas stained to reveal pattern of transduction obtained following neural tube delivery of 0.5 μl of EF1α-PLAP virus (10^9^ TUs/ml). The percent transduction for the left (left panel) and right (right panel) eyes of two of the three animals analyzed is shown. (C) FAR SUP region of a treated retina immunostained for GFP. Arrows indicate staining in photoreceptor cell bodies. GFP staining is also visible in the inner segments and outer segments of these cells. GCL, ganglion cell layer; INL, inner nuclear layer; IPL, inner plexiform layer; IS, inner segments; ONL, outer nuclear layer; OS, outer segments. (D) Topographical distribution of GFP-expressing cells in treated right eyes from treated animals 2 (left) and 1 (right). The percent transduction of each of the four retinal regions was plotted on the *z*-axis as a function of location along the superior–inferior axis of the eye (*x-*axis) and distance from the midline (*y*-axis). These analyses represent the results obtained from serial sections over 500 μm beginning at the midline axis and moving laterally.

The distribution of transduced cells within treated retinas was determined by examining serial sections of the right eye cup taken along the vertical meridian (Figure 5). Each section was divided into four contiguous regions, and the percent of retinal cells positive for GFP was estimated for each region (Figure 5A). In all retinas examined, greater than 90% of the GFP staining was localized to the photoreceptor cell layers (Figure 5C). The estimates of percent GFP staining observed in each 14-μm section were used to create a topographical map of the percent GFP staining observed within the 500-μm region for each animal. The results obtained for two of the six treated animals (animals 1 and 2; Figure 5D) illustrate the variation observed in the distribution of transduced cells in the 500-μm region selected for these analyses. The estimates for the number of integrated transgenes per genome for animals 1 and 2 were 0.08 and 0.07, respectively. The spatial distribution of transduced cells observed in the retinas of these animals was similar to that observed in our analyses of flat-mounted EF1α-PLAP-treated retinas (Figure 5B). Based on the percent transduction estimates obtained for retinas treated with the EF1α-PLAP virus, a conservative estimate for the percent transduction of the retinas treated with the EF1α-GC1-IRES-eGFP virus would be 15%–40% of the photoreceptor population, with the actual value depending on the quality of the embryonic injection.

### Morphometric Analyses Suggest That Lentiviral Treatment Slows Retinal Degeneration

**Figure 6:**
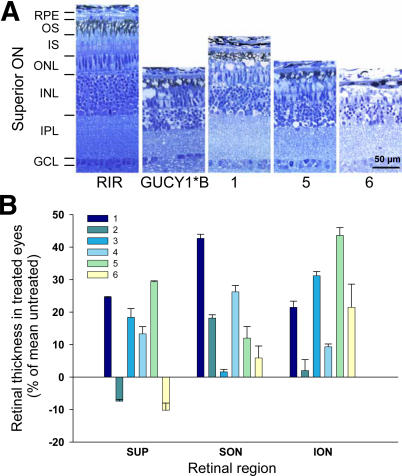
Comparison of Retinal Morphology of Wild-Type RIR, Untreated GUCY1*B, and Treated GUCY1*B Chickens (A) The morphology of the retinas of treated animals was examined to determine if the viral treatment had affected the course of retinal degeneration. Regions were analyzed along the vertical meridian of the right eyes of experimental and control animals; representative micrographs from the locus superior to the optic nerve (SUP ON) are shown from one wild-type, one untreated GUCY1*B, and three treated retinas (animals 1, 5, and 6). GCL, ganglion cell layer; INL, inner nuclear layer; IPL, inner plexiform layer; IS, inner segments; ONL, outer nuclear layer; OS, outer segments; RPE, retinal pigment epithelium. (B) Relative percent change in retinal thickness of treated animals. Retinal width (in micrometers) from the outer limiting membrane to the ganglion cell layer was measured at six loci (100 μm apart) within the regions examined. These widths were expressed as percent change relative to average widths of the untreated GUCY1*B retinas in these regions, which were set to zero for graphing purposes. A simple one-sample *t*-test (null hypothesis that treatment groups are not different from untreated GUCY1*B) was used to analyze the results. The results of this test showed that retinal thicknesses of the treated animals in the SUP ON (SON) and INF ON (ION) regions were greater than those of untreated animals (*p* < 0.05). Four of six treated animals also showed evidence of slowing of degeneration in the SUP region, but this was not statistically significant. The key for animal number is shown in the upper left of the graph.

The histopathology associated with the absence of GC1 in the GUCY1*B retina has been well-documented [12,47]. Degeneration first appears 7–10 d post-hatch and is limited to the photoreceptor cells. By 21 d of age, the number of identifiable outer segments is significantly reduced, and at 115 d of age, very few photoreceptors remain in the central retina and gross pathological changes are present in the retinal pigment epithelium. By 6–8 mo, degenerative changes have progressed to peripheral regions of the retina and little remains of the photoreceptor cell layer. Sections of retinas of the right eyes of treated animals were compared to those of age-matched wild-type and GUCY1*B untreated animals to determine if the viral treatment altered the histopathology. The results suggested that treatment may have slowed retinal degeneration in some animals but did not prevent degeneration (Figure 6A). The best preservation of the retina was observed in animal 1, as evidenced by increased numbers of nuclei within the outer nuclear layer and increased thickness of the inner plexiform layer relative to untreated GUCY1*B retina. In contrast, animal 6 had severe loss of cells from the outer nuclear layer across all regions examined, and the retinal pigment epithelium had changes characteristic of the late-stage degeneration usually observed in untreated animals [12]. The retinal changes were quantified by measuring the distances between the outer limiting membrane and the ganglion cell layer within each of the four regions sampled. These measures were expressed as percent change in retinal thickness relative to that observed in untreated GUCY1*B retinas (Figure 6B). The results of these analyses revealed that the thickness of all of the treated retinas relative to untreated retinas was significantly greater in the SUP ON (*t*[5] = 2.9, *p* < 0.05) and INF ON (*t*[5] = 3.5, *p* < 0.05), the two regions of the retina that normally undergo the most rapid degeneration in untreated GUCY1*B retinas [12]. The SUP region of the retinas of four of the six treated animals also showed signs of slowed degeneration, but these changes were not found to be significant. Analyses of the FAR SUP region revealed that one treated animal showed signs of unusually severe retinal degeneration in this region; this region in the remaining five treated animals was not significantly different from that of untreated retinas (data not shown).

## Discussion

### Expression of GC1 in GUCY1*B Photoreceptors Restores Cell Function and Vision

This study is the first to our knowledge to demonstrate restoration of sight in an animal model of LCA1. Using a lentiviral vector system and an *in ovo* approach, we successfully delivered normal copies of GC1 transgenes to retinal cells of GUCY1*B chickens, the expression of which was sufficient to restore function to photoreceptor cells as measured by ERG. Importantly, the transduced photoreceptors supported robust visual behavior in treated animals. In the six treated animals exhibiting sighted behavior, the ERG, retinal morphology, and integrated viral-transgenes-per-genome data were indicative of overall treatment efficacy. Larger ERG responses, better preserved retinal morphology, and higher integrated transgene numbers were observed in animals that received the highest visual performance scores, while the absence of ERG responses, poor retinal morphology, and low integrated transgene numbers were associated with animals exhibiting poor visual performance.

### Achieving Long-Term Vision Restoration in LCA1

Gene-based therapies of autosomal recessive retinal degenerative diseases are based on the seemingly simple principle that expression of a viable copy of a defective gene will produce functional protein, restore function to disabled cells and tissues, and prevent degeneration. Our therapy successfully addresses the first two goals but falls short of preventing retinal degeneration. Slowed but progressive retinal degeneration has also been observed in other successful gene transfer experiments for photoreceptor disease (for examples see [16,18,48]). To produce an effective, long-term therapy, degeneration must be prevented or limited.

The patchy transduction pattern generated by our treatment (Figure 5B) leads to an intermingling of nontreated, dysfunctional photoreceptor cells with treated, functioning cells. Thus, the phenomenon known as the “bystander” effect, or nonautonomous cell degeneration, may be at play [49,50]. In many inherited photoreceptor diseases linked to genes whose expression is limited to rod cells, cone photoreceptor cells also degenerate. The retinal degeneration patterns observed in aggregation embryos created from normal and mutant mice expressing mutant rhodopsin (Pro347Ser) [51] and in hemizygous transgenic female *rds* ^−/−^ mice carrying a transgene encoding normal rds/peripherin on the X-chromosome [52] further illustrate this phenomenon. Degeneration of photoreceptors expressing abnormal proteins had a negative impact on the ability of otherwise healthy cells to survive, a trend that was tempered in retinas containing higher percentages of normal cells. If the ratio of nontreated to treated cells is high in our animals, then degeneration of the nontreated cells could be adversely affecting survival of the treated cells. The simplest approach to achieve long-term restoration of sight in our paradigm may be to increase the total number and/or density of photoreceptor cells transduced by the viral vector. By increasing the titer of the injected virus from 10^9^ to 10^10^ TUs/ml, for example, we can effectively increase the percent transduction of the photoreceptors from approximately 40% to 85% [33].

It is unclear what percent of the total photoreceptor cell population must be transduced to overcome the phenomenon of nonautonomous, density-dependent photoreceptor degeneration. The results obtained in this study and in other studies of inherited retinal disease suggest that there may be a “critical mass” of photoreceptor cells that must be preserved to obtain long-term cell survival. Two factors that seem to influence whether a photoreceptor cell lives or dies are the percent of the total photoreceptor population slated for death and the topographical position of that cell relative to the degenerating cells. For example, in the GC1 knockout mouse, degeneration of the cones, which represent 3%–5% of the photoreceptor population and are distributed evenly across the retina, does not adversely affect survival of the rod cells [53,54]. In humans with rod-specific retinal degeneration caused by rhodopsin gene mutations, cone cell function and survival becomes compromised when greater than 75% of the affected rod cells degenerate [55]. In human cone dystrophies, there can be an impact of degenerating cone cells on rod cell survival, most evident in the central retina, wherein both photoreceptors peak in density and are closely adjacent [56,57]. The notion that therapies producing large contiguous regions of treated cells aid survival of the treated cells gains support from the success of subretinal delivery of viral vectors in studies of mouse [21,24] and dog [20,58] models of LCA2, a disease caused by mutations in the gene encoding RPE65. In these studies, the retinal pigment epithelium cells that normally express RPE65 are the primary targets of therapies designed to deliver normal copies of RPE65 to these cells. Since each retinal pigment epithelium cell supports the function of many photoreceptor cells, successful treatment of the retinal pigment epithelium effectively treats large, contiguous regions of photoreceptor cells. Other studies using subretinal delivery to treat primary photoreceptor diseases have not been able to entirely halt progressive degeneration [16,18,48]. Although we were able to restore vision by transducing a relatively low percent of the photoreceptor population, transduction of a larger percent of these cells may lead to significant improvement in the long-term effectiveness of our gene therapy.

Another modification to our therapeutic strategy that may improve treatment efficacy is use of photoreceptor-specific promoters to drive transgene expression in our viral vector. Limiting expression of GC1 to photoreceptors could improve treatment effectiveness by eliminating any untoward effects induced by ubiquitous GC1 expression. A future strategy might also include bicistronic therapeutic transgenes that not only encode GC1 but also encode factors that improve photoreceptor viability or prevent cell death. Several members of the fibroblast growth factor family [59,60], ciliary neurotrophic factor [61], and the apoptotic inhibitor bcl-2 [62,63] have been shown to delay photoreceptor degeneration in models of inherited retinal disease. Recently, a factor secreted by rod cells has been identified that appears to support cone cell viability [64].

### Clinical Relevance to LCA1

The present proof-of-concept experiments in the avian model of LCA1, albeit with an *in ovo* approach, prompts thoughts of translational studies en route to human clinical trials of gene transfer. The observation that the photoreceptor transduction efficiencies achieved in this study support near normal visual behavior in the treated animals is encouraging and consistent with what is well-known clinically about human retinal degeneration diseases. Despite major deficits in retinal function, as measured by visual fields or ERGs, patients can display serviceable vision if even small islands of functioning retina are retained [65–67]. Thus, it is reasonable to expect that restoration of useful vision in LCA1 patients would not require treatment of the entire retina.

Based on human postmortem studies of retinal histopathology in an 11.5-y-old patient with LCA1 caused by *GUCY2D* mutation [13], in utero gene transfer may not be the only option in this life-altering but nonfatal ocular disease. This clinical–pathological analysis showed that there were regions with preserved retina cells despite profound visual disturbance at this age. Early treatment of pediatric LCA1 patients, however, would seem worth considering because of a report of prenatal retinal degeneration in this genotype [68]. Although serious visual consequences are characteristic of molecularly defined LCA1 [14,69–71], there are only limited data on the retinal pathology underlying the major dysfunction in LCA1 [13]. In vivo optical scanning techniques have advanced sufficiently to permit quantification of the retinal micropathology present in LCA1 patients with different *GUCY2D* mutations. Such studies would be a prerequisite for specific planning of future trials of treatment in humans [72].

Finally, the safety and efficacy of virus-based gene transfer is also a major point of consideration for future studies intent on developing a genetically based treatment for LCA1 in humans. To date, several different types of virus-based vectors have been engineered for effective gene transfer in vivo. In this study, we chose to use a lentiviral vector system. Lentivectors efficiently and stably transduce retinal progenitor cells, a characteristic that made them particularly useful in our *in ovo* treatment paradigm. In considering treatments for human LCA1, it will be important to adopt the vector system that is best suited for the treatment situation. For example, vectors based on adeno-associated virus have proven to be efficient vehicles for gene transfer to retinal photoreceptors [73–75] and hold promise for clinical trials of retinal disease in humans [72]. Lentiviral vectors also exhibit great promise as vehicles for gene therapy [76,77]. A recent study showing that nonintegrating lentiviral vectors are able to restore retinal function to a mouse model of LCA2 [78] significantly increases the safety and value of this vector for human gene therapy applications.

## Supporting Information

Video S1 Optokinetic Behaviors of Wild-Type RIR and Untreated GUCY1*B Chickens Optokinetic behavior was recorded for wild-type RIR and untreated GUCY1*B chickens at 21 d of age. High-contrast vertical square wave gratings with spatial frequencies of 0.065 or 0.26 cycles·degree^−1^ (bar widths of 5 cm and 1.25 cm, respectively) were used to elicit responses from the RIR chicken. Both stimuli induced reflexive head movement characteristic of the optokinetic response in birds. The 5-cm stimulus was used in tests of the untreated GUCY1*B chicken. No optokinetic responses were elicited in this animal.
(4.4 MB MOV)

Video S2 Volitional Visual Behaviors of Wild-Type RIR and Untreated GUCY1*B Chickens The visual behaviors of the wild-type RIR chickens were recorded at 7 d of age, and those for the untreated GUCY1*B chicken were recorded at 7 and 21 d of age. The wild-type RIR chickens oriented to flags and novel stimuli within their visual field but avoided them. Visually directed pecking was most strongly elicited by food. Untreated GUCY1*B chickens did not attend to moving stimuli within their visual fields. They remained relatively stationary but when they moved they tended to do so in circles. When the animals were stationary, slow nystagmiform movements of the head, which are one of the identifying stereotypic behaviors exhibited by these animals, were observed.
(6.8 MB MOV)

Video S3 Optokinetic Behavior of Treated GUCY1*B Animal 2 The optokinetic responses of animal 2 were recorded at 13 d of age. The vertical square wave grating with a spatial frequency of 0.26 cycles·degree^−1^ (bar width of 1.25 cm) was used to elicit responses from animal 2. When placed within the rotating stimulus drum, animal 2 exhibited reflexive head movements characteristic of the optokinetic response.
(2.4 MB MOV)

Video S4 Volitional Visual Behaviors of Treated GUCY1*B Animal 2 The visual behavior of animal 2 was recorded at 3, 7, and 13 d of age. On days 3 and 7, animal 2 pecked at black printed dots on white paper (1, 2, 4, and 10 mm diameter), visually followed a black dot centered on a white flag, and pecked at shiny objects. On day 13, animal 2 repeatedly ran toward a member of the investigative team and pecked at shiny objects. The rapid, directed locomotion exhibited by animal 2 was never observed in untreated GUCY1*B animals and may have been a manifestation of behavioral imprinting to the investigator who raised it.
(8 MB MOV)

## Abbreviations

E[number]: embryonic day [number]
ERG: electroretinogram
FAR SUP: far superior
GC1: guanylate cyclase–1
GCAP1: guanylate cyclase activating protein–1
GFP: green fluorescent protein
INF ON: inferior optic nerve
IRES: internal ribosome entry site
LCA: Leber congenital amaurosis
LCA[number]: Leber congenital amaurosis type [number]
OKN: optokinetic nystagmus
PBS: phosphate-buffered saline
PDE: phosphodiesterase
retGC1: retinal guanylate cyclase–1
RIR: Rhode Island Red
RT: reverse transcription
SEM: standard error of the mean
SUP ON: superior optic nerve
SUP: superior
TU: transducing unit
